# Non-iterative Directional Dark-field Tomography

**DOI:** 10.1038/s41598-017-03307-6

**Published:** 2017-06-12

**Authors:** Florian Schaff, Friedrich Prade, Yash Sharma, Martin Bech, Franz Pfeiffer

**Affiliations:** 1 Lehrstuhl für Biomedizinische Physik, Physik-Department & Munich School of BioEngineering, Technische Universität München, 85748 Garching, Germany; 2 Computer Aided Medical Procedures, Technische Universität München, 85748 Garching, Germany; 3 Medical Radiation Physics SUS, Lund University, 22185 Lund, Sweden; 4 Institut für diagnostische und interventionelle Radiologie, Klinikum rechts der Isar, Technische Universität München, 81675 München, Germany

## Abstract

Dark-field imaging is a scattering-based X-ray imaging method that can be performed with laboratory X-ray tubes. The possibility to obtain information about unresolvable structures has already seen a lot of interest for both medical and material science applications. Unlike conventional X-ray attenuation, orientation dependent changes of the dark-field signal can be used to reveal microscopic structural orientation. To date, reconstruction of the three-dimensional dark-field signal requires dedicated, highly complex algorithms and specialized acquisition hardware. This severely hinders the possible application of orientation-dependent dark-field tomography. In this paper, we show that it is possible to perform this kind of dark-field tomography with common Talbot-Lau interferometer setups by reducing the reconstruction to several smaller independent problems. This allows for the reconstruction to be performed with commercially available software and our findings will therefore help pave the way for a straightforward implementation of orientation-dependent dark-field tomography.

## Introduction

Grating-based X-ray phase-contrast imaging (GBI) is an interferometry technique developed a decade ago. In addition to the conventional attenuation image, two additional contrast modalities are acquired simultaneously^1–4^: the differential phase and dark-field contrast. While the widely used X-ray attenuation imaging purely relies on the reduction of the intensity of X-rays when they pass through an object, the differential phase-contrast image is based on the refraction of X-rays. Phase contrast imaging can be several orders of magnitude more sensitive to changes within an object than attenuation-based imaging^1^. The additional dark-field contrast is interpreted as scattering of X-rays by structures of sizes below the spatial resolution of the imaging system^3, 5–10^. Because of its ability to combine scattering information with spatial resolution in a single image, dark-field imaging is particularly useful for the investigation of microscopic changes inside large objects. Just like conventional X-ray imaging, GBI is not limited to radiography only, but volumetric information an be obtained using computed tomography (CT) for both the phase-contrast^4, 11–16^ and the dark-field signals^17–22^.

A conventional grating-interferometer used for GBI consists of parallel grating lines oriented in a certain direction. The grating-interferometer is therefore sensitive only to phase gradients and scattering information perpendicular to the grating lines. The anisotropic sensitivity of a grating-interferometer can be used to characterize the orientations of microscopic scattering structures both in 2-D^23–25^, and 3-D^20, 26–29^. For the three-dimensional case, a complete reconstruction of the anisotropic scattering distribution has only been achieved by the use of complex reconstruction techniques so far^26, 27, 29^. As a three-dimensional scattering distribution is reconstructed in each voxel, oriented dark-field tomography is also known as X-ray Tensor Tomography (XTT). The data acquisition for XTT, as presented by Vogel *et al*.^29^ and Malecki *et al*.^26^ requires highly specialized, bulky and expensive hardware. Moreover, the reconstruction of three dimensional scattering information in every voxel requires the use of computationally intensive iterative reconstruction methods. Even though methods to reconstruct vector fields from projection data exist e.g. for magnetic resonance imaging^30^, acoustic tomography^31^ or X-ray velocimetry^32^, they cannot be directly used for GBI due to differences in the underlying physics and acquisition mechanisms.

In a specialized case where structures in only one particular orientation are of interest, the one-dimensional sensitivity of a grating set-up with horizontal gratings (in the following we assume the rotation axis to always be vertical) can be used e.g. to identify misaligned fibres in a carbon fibre reinforced material^20^. However, for phase-contrast tomography it is advantageous that the orientation of the grating lines coincides with the axis of rotation. Thus, nearly all works so far used a vertical alignment of both gratings and rotation axis; for phase-contrast CT^4, 11–16^ as well as for dark-field CT^18, 19, 21, 22^.

In this paper, we present a way to obtain results equivalent to XTT by directly measuring several different scattering orientations within an object. In contrast to XTT, our proposed method does not require specialized acquisition hardware and is fully compatible with conventional CT stages. For XTT, a sample is rotated around three rotation axes and the combined signal is later split into auxiliary scattering orientations during reconstruction. Our proposed method directly measures several distinct scattering orientations by the use of horizontal gratings and each individual orientation is reconstructed separately using filtered back-projection (FBP). On the software side, the main focus of this work is to shift away from the complex reconstruction algorithm used for XTT to more accessible, commercially available and well-established reconstruction and image processing techniques. We use the concept of rotational invariance to show that a horizontal arrangement of the gratings is superior to a vertical one for arbitrarily scattering samples^33^. Results of an orientation dependent dark-field tomography obtained with the proposed method are presented and compared to those obtained from an XTT reconstruction with the same raw data.

## Results and Discussion

**Figure 1:**
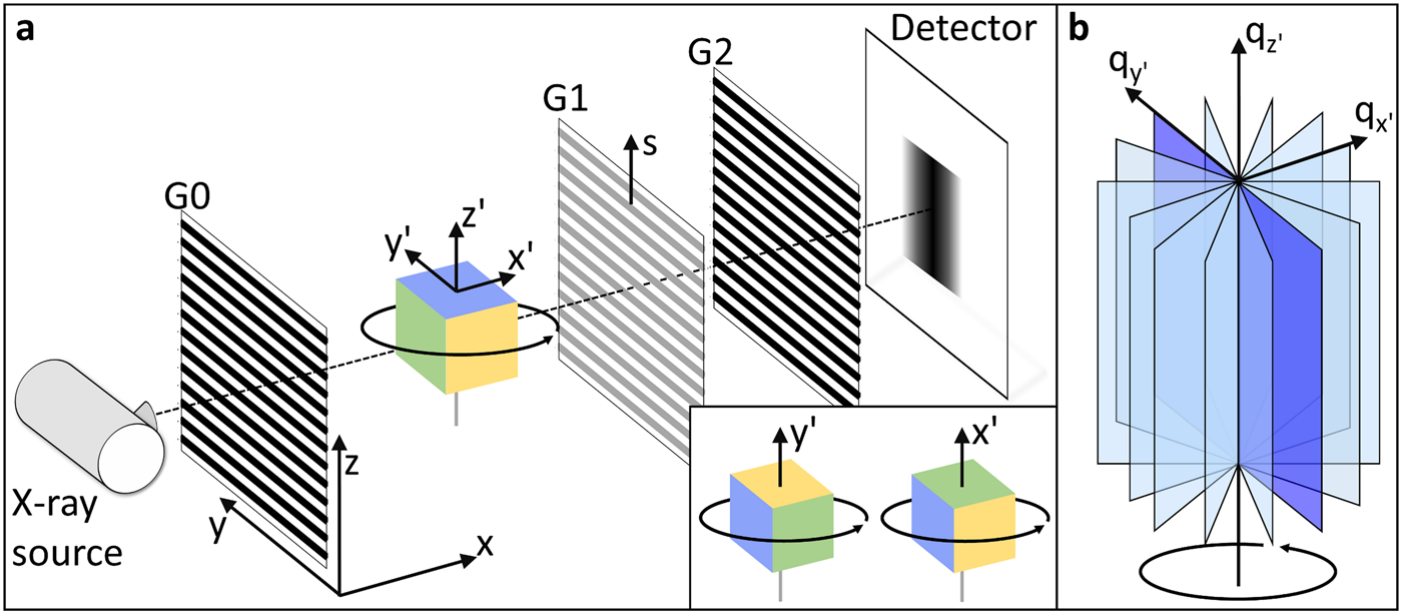
Talbot-Lau X-ray grating interferometer with horizontal gratings. (**a**) Schematic set-up of a three-grating interferometer including a source grating G0, a phase-grating G1 and an analyzer grating G2. During a tomographic measurement the samples coordinate system (x’, y’, z’) is rotated with respect to the laboratory coordinate system (x, y, z). The sensitivity direction of the grating interferometer is given by ${\bf{s}}$. (**b**) For each sample orientation, the dark-field signal probes a specific two-dimensional slice of the three-dimensional reciprocal space used to describe small-angle scattering. The signal in the scattering direction parallel to the rotation axis, z’ in this case, is rotationally invariant. Inset: A rotation of the sample can be used to align different scattering directions parallel to the rotation axis.

A Talbot-Lau X-ray grating interferometer consists of three gratings, as shown in Fig. 1a (For a complete description of such a grating setup, please refer to e.g. Pfeiffer *et al*.^5^).

The origin of the dark-field signal is closely related to small-angle X-ray scattering (SAXS), which is usually described in reciprocal space^5, 9, 10^. Each scattering orientation is denoted by a momentum transfer vector **q**, which have a certain scattering intensity for each voxel within the investigated object. In GBI, the relevant scattering angles are sufficiently small to fall within the regime of small-angle approximation. It follows that for a projection image only a two-dimensional slice of the three-dimensional reciprocal space perpendicular to the X-ray propagation direction is relevant for the dark-field signal, as shown in Fig. 1b. Using the convention presented in Fig. 1, a projection image taken along the $x^{\prime}$-axis of the sample consists of information only from scattering vectors from the ${q}_{y^{\prime} }$, ${q}_{z^{\prime} }$-plane. The well-defined sensitivity of the grating interferometer, denoted as ${\bf{s}}$ in Fig. 1a, further restricts the recorded scattering information to **q**-vector components parallel to ${\bf{s}}$ ^9^.

During a computed tomography measurement, the sample is rotated around the $z$-axis, as indicated for the central cube in Fig. 1. This causes a different two-dimensional plane of the reciprocal space to be probed in every projection. Additionally, the orientation of ${\bf{s}}$ in sample coordinates, i.e. the primary scattering direction of the dark-field signal, denoted as ***ε***, may change. Such a rotation by an angle $\theta$ is described by a rotation matrix ${R}_{z}$:

1 $${{\bf{R}}}_{{\bf{z}}}=(\begin{array}{ccc}\cos \,\theta  & -\,\sin \,\theta  & 0\\ \sin \,\theta  & \cos \,\theta  & 0\\ 0 & 0 & 1\end{array})$$

It is clear that vectors with only a $z-$ component (or ${q}_{z}$ in reciprocal space) are unaffected by a rotation around the $z$-axis:

2 $${{\bf{R}}}_{{\bf{z}}}\cdot (\begin{array}{c}0\\ 0\\ z\end{array})=(\begin{array}{c}0\\ 0\\ z\end{array})$$

Therefore, a constant relation between **s** and ***ε*** is maintained throughout a CT scan when **s** is parallel to the rotation axis, i.e. ${\bf{s}}=(\mathrm{0,}\,\mathrm{0,}\,1)$ here. For this, the grating bars need to be aligned perpendicular to the rotation axis^7, 20^. With the rotation axis for laboratory set-ups generally aligned vertically, we call this grating alignment horizontal. This configuration allows us to unambiguously assign a single primary scattering direction, ***ε***, to a CT-scan. Any arbitrary scattering direction ***ε*** can be set parallel to the rotation axis by positioning the sample accordingly, as shown in the inset of Fig. 1a.

One important criterion for a correct tomographic reconstruction is the rotational invariance of the measured signal^33^. The standard mathematical description requires that the sum of the line-integrals of the signal is constant under rotation. In other words, only the spatial distribution of the recorded signal should change, but not its total strength. In order to fulfil this condition for dark-field tomography, the recorded signal should ideally arise from the same $q^{\prime}$-vectors for all projections. Given the way that the dark-field signal is formed in a grating interferometer^9^, $q^{\prime}$-vectors not exactly parallel to ${\bf{s}}$ also contribute to the dark-field signal. Owing to this, perfect rotational invariance can not be achieved in dark-field imaging. However, the largest contribution to the dark-field signal is from vectors parallel to the sensitivity axis. A horizontal arrangement of the gratings therefore provides the best approximation of rotational invariance for the dark-field signal.

### Horizontal vs. vertical grating alignment

**Figure 2:**
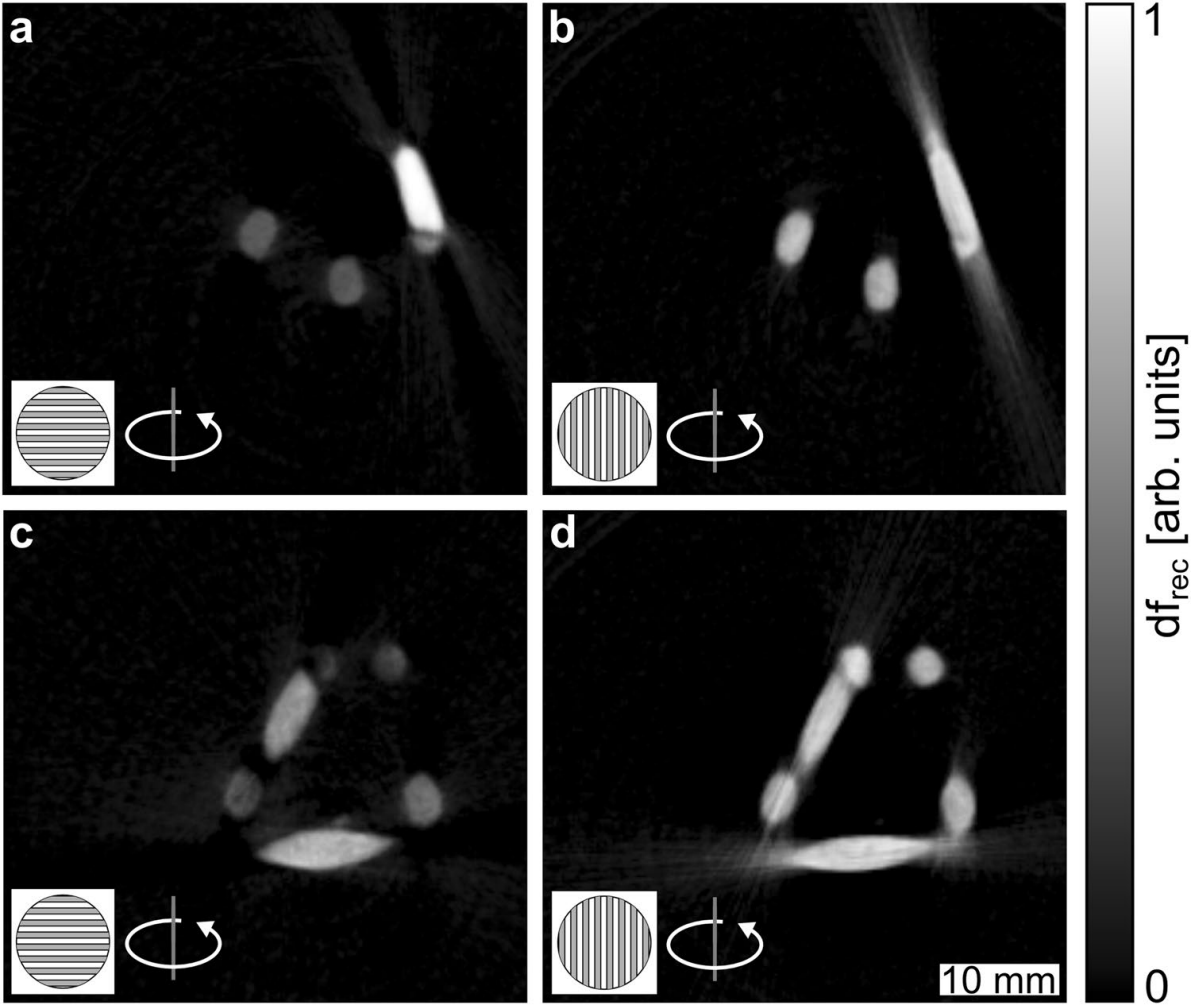
Comparison of vertical and horizontal grating reconstructions. The same sample was measured and reconstructed twice with a change in grating orientation. The reconstructed dark-field signal, $d{f}_{rec}$, is compared in two slices at different heights of the sample, shown in (**a**,**c** and **b**,**d**) for horizontal and vertical gratings, respectively. (**b**,**d**) The reconstruction results for vertical gratings are affected by severe streak artefacts, caused by strongly aligned fibres within the sample. (**a**,**c**) With a horizontal alignment of the gratings these artefacts are significantly reduced, owed to the improved rotational invariance of the scattering data. All reconstructions were performed with simple filtered back-projection.

In order to demonstrate the advantage of horizontal gratings, we measured an approximately 2 × 2 × 2 cm^3^ large sample consisting of several wooden toothpicks glued together to form the letters “TUM”. This phantom is characterized by areas with highly oriented wooden fibres in multiple directions. Furthermore, its rather simple geometrical shape allows for a clear visualization. We compare two full dark-field CT scans, with the only difference being the alignment of the gratings. The datasets were reconstructed using filtered back-projection and we obtained one volume with vertical, ${V}_{{\rm{v}}}$, and one volume with horizontal grating alignment, ${V}_{{\rm{h}}}$. Slices of the reconstructed volumes are shown in Fig. 2. The reconstructed dark-field signal, $d{f}_{rec}$, at the same position of the sample is shown for ${V}_{{\rm{h}}}$ and ${V}_{{\rm{v}}}$ in panels (a) and (c), respectively. A second slice at a different position is given in panels (c) (${V}_{{\rm{h}}}$) & (d) (${V}_{{\rm{v}}}$). Note that parts of the reconstructed slices appears dissimilar between horizontal and vertical grating alignment owing to the different scattering information recorded between the two CT scans. It is evident that ${V}_{{\rm{v}}}$ suffers from strong streak artefacts. The reconstruction of ${V}_{{\rm{h}}}$ is nearly free from such artefacts.

We attribute the artefacts seen in ${V}_{{\rm{v}}}$ to the anisotropic nature of the scattering signal from wooden fibres. The scattering signal acquired with vertical grating alignment is perpendicular to a rotation about the *z*-axis. Therefore, the probed scattering orientation differs for each projection and consequently the dark-field signal from each point of the sample depends on the rotation of the sample. Filtered back-projection relies on a constant signal from a single point of the sample under rotation. It is clear that this is not the case for ${V}_{{\rm{v}}}$. As varying signals for different projections cannot be compensated for in FBP, artefacts such as the ones present here are likely to appear. In particular, fibres in the imaging (*x*-*y*-) plane give rise to a strong scattering signal when aligned in the X-ray beam direction. With a vertical grating alignment, a rotation of 90° causes this signal to vanish almost entirely. In contrast, the signal acquired with horizontal gratings does not change nearly as much under rotation, resulting in considerably fewer artefacts in the reconstruction. Owing to off-axis scattering, i.e. contributions of $q^{\prime}$-vectors not exactly parallel to ${\bf{s}}$, minor artefacts remain to be seen in ${V}_{{\rm{h}}}$. However, their magnitude is negligible compared to the rest of the reconstruction. Hence, we conclude that horizontal gratings are advantageous for dark-field tomography of samples containing unknown fibre orientation.

### Registration-based dark-field tensor tomography

**Figure 3:**
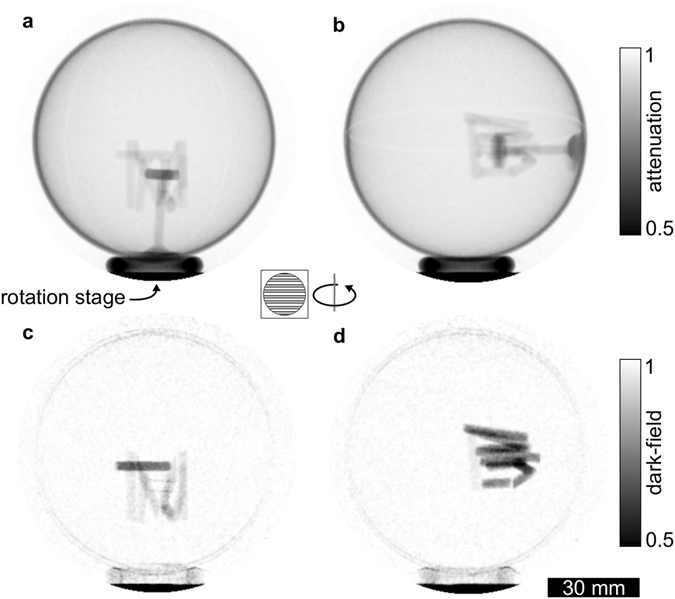
Sample rotation during the measurement. In order to allow for an arbitrary sample positioning on top of the rotation axis, the sample was mounted inside a hollow sphere. This auxiliary sample mounting allows to position any axis through the sample parallel to the sensitivity direction of the gratings. Attenuation (**a**) and (**b**) as well as dark-field (**c**) and (**d**) projections are shown for two positions of the sample.

An immediate consequence of the preceding considerations is that it is insufficient to measure the sample only in one position with respect to the gratings. The full voxel-wise three-dimensional scattering distribution can only be obtained by probing multiple scattering orientations. With this in mind, we measured a full tomography of the phantom for several different sample orientations such that for every acquisition we probe a unique scattering orientation ${{\boldsymbol{\varepsilon }}}_{k},\,k=\mathrm{1,}\ldots ,\,7$. In other words, for every sample position $k$, the scattering direction ***ε***_*k*_ satisfies the condition of rotational invariance. For all measurements, the rotation axis and grating alignment remained unchanged. The only change between the scans was the orientation of the sample on top of the rotation stage, which was performed by hand. The problem of allowing for arbitrary sample positioning was solved by using an auxiliary sample holder, as demonstrated in Fig. 3. Attenuation (a) and (b), as well as dark-field (c) and (d) projections for two different ***ε***_*k*_ are shown. The sample is mounted inside a hollow sphere, which allows for a free rotation in between the individual measurements. As a consequence, no limitations exist for which ***ε***_*k*_ can be probed. As the sphere consists only of a thin layer of plastic, it does not contribute significantly to the dark-field and attenuation signals. Furthermore, any minor contribution of the sphere is separated from the sample by the CT reconstruction. In order to sample the unit sphere of possible scattering orientations sufficiently, the ***ε***_*k*_ were chosen to approximately sample three orthogonal directions, as well as the four corresponding space diagonals. Since all seven measurements probe a different scattering orientation, the resulting datasets are completely independent from one another. The attenuation and dark-field volumes, ${V}_{k,{\rm{a}}}$ and ${V}_{k,{\rm{df}}}$, were reconstructed using filtered back-projection for all seven datasets.

**Figure 4:**
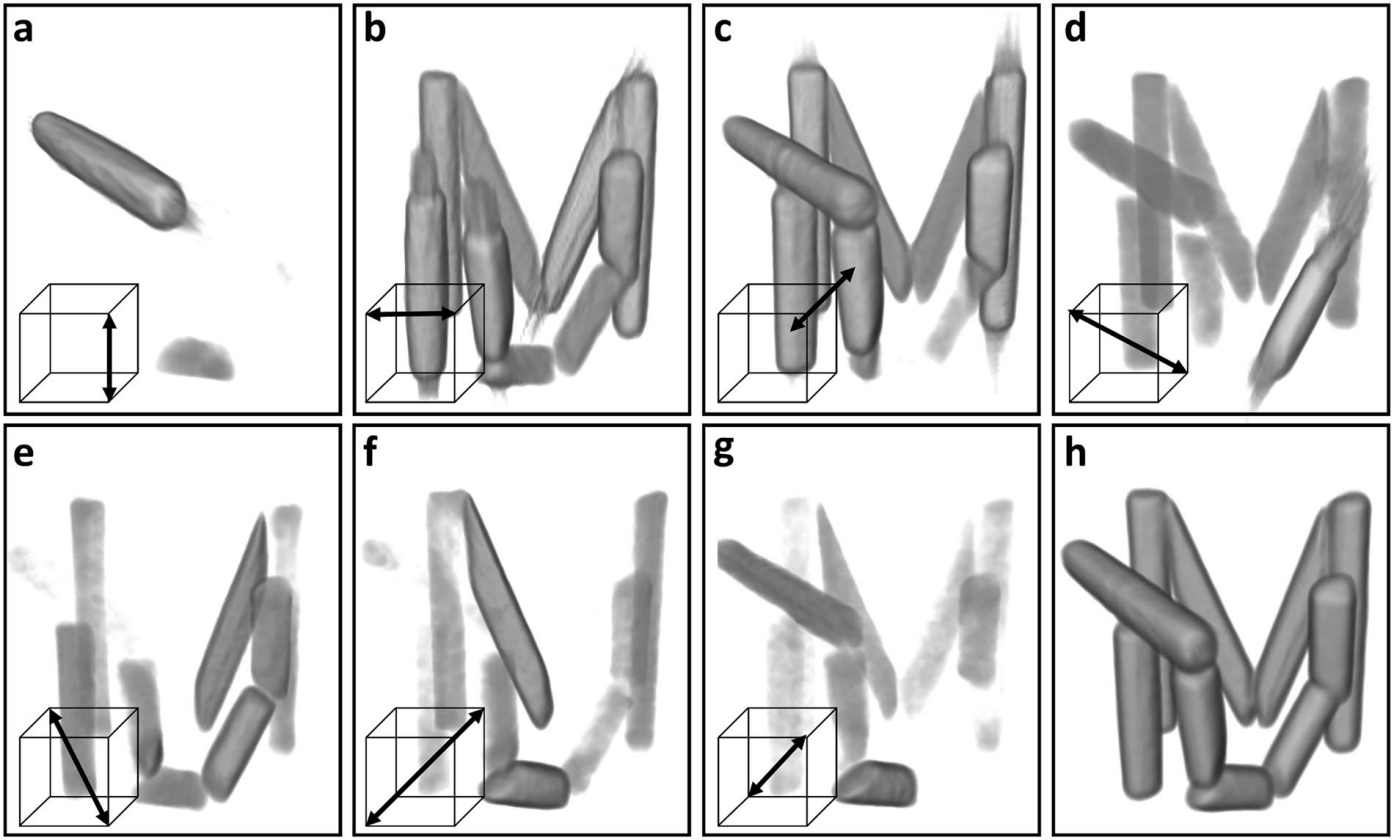
Reconstructions with different sensitivity directions. (**a**–**g**) Volume renderings of the reconstruction results for seven individual datasets after registration. For each measurement, the sample was rotated around a different axis, as indicated by the arrow in the insert. These axes were always kept perpendicular to the grating lines so a simple filtered back-projection reconstruction could be used. (**h**) The sum of all dark-field reconstructions.

Since the attenuation and dark-field signals for each of the seven datasets were acquired simultaneously in the GBI setup, they are intrinsically registered. However, the different measurements are rotated and translated with respect to each other. Considering ${V}_{\mathrm{1,}{\rm{a}}}$ as the reference dataset, the remaining six datasets were registered to the reference using the isotropic attenuation signal ${V}_{k,{\rm{a}}};\,k=\mathrm{2,}\ldots ,\,7$. The registration procedure yields a rotation matrix ${{\bf{R}}}_{{\bf{k}}}$, which describes the rotation of each volume with respect to the reference. Volume renderings of all seven reconstructed and registered dark-field volumes ${V}_{k,{\rm{df}}}$ are shown in Fig. 4a–g. Within each panel, the approximate orientation of the probed scattering component is indicated by an arrow. The complementarity of the information recorded in the individual volumes can be seen clearly. Depending on the relative orientation of the wooden fibres and reconstructed scattering component, different parts of the sample are visible in different volumes. As the probed scattering orientations are well distributed on the unit hemisphere, a mean dark-field signal ${V}_{\overline{{\rm{d}}{\rm{f}}}}=\frac{1}{7}{\sum }_{k=1}^{7}{V}_{k,{\rm{d}}{\rm{f}}}$ was calculated from the seven volumes. A rendering of the resulting mean dark-field reconstruction is shown in Fig. 4h.

In order to yield information about structural orientation, it is important to know the scattering orientations that are probed in every volume ${V}_{k,{\rm{df}}}$ after registration. Assuming that the scattering orientation probed in ${V}_{\mathrm{1,}{\rm{df}}}$ is ${{\boldsymbol{\varepsilon }}}_{1}={(\mathrm{0,}\mathrm{0,}1)}^{{\rm{T}}}$ i.e. the sensitivity of the GBI setup, the probed orientation of the other six volumes can be calculated as ${{\boldsymbol{\varepsilon }}}_{k}={{\bf{R}}}_{k}{(\mathrm{0,}\mathrm{0,}1)}^{{\rm{T}}};\,k=\mathrm{2,}\ldots ,\,7$. The resulting vectors correspond to the orientation of the scattering components reconstructed in each volume after registration. The seven volumes along with the corresponding orientations can be used to reconstruct a scattering tensor at every voxel as is done for XTT. In XTT, several scattering components are being reconstructed simultaneously into seperate volumes^26, 29^. Here, we measured each of the scattering components directly.

### Comparison with XTT

We took all projections recorded for the direct FBP-based reconstruction and fed them into the XTT reconstruction algorithm as described by Vogel *et al*.^29^ We set the algorithm to reconstruct the exact same seven components that were directly probed with horizontal gratings. The major differences between the two approaches is that in XTT, we use all the projections to reconstruct auxiliary scattering orientations while in the FBP based approach we independently reconstruct the orientations from distinct subset of projections.

**Figure 5:**
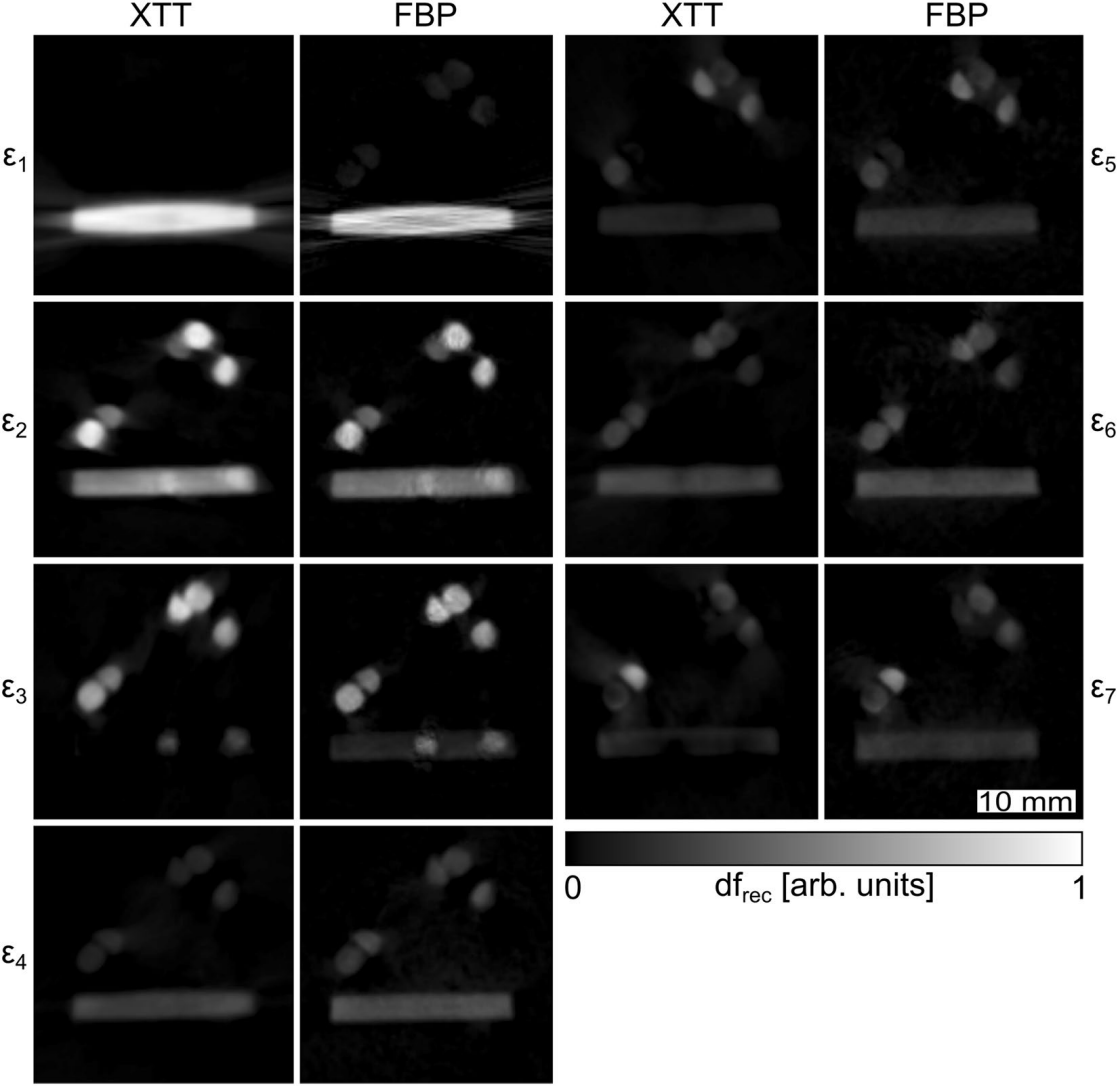
Comparison of scattering components reconstructed with XTT and direct FBP based measurement. The same scattering components ***ε***_*k*_ were reconstructed with both methods and are shown for a single slice. The columns labelled XTT and FBP show the results from an XTT reconstruction and the results directly measured with horizontal gratings and reconstructed using FBP, respectively. The greyscale for all images is the same. An animation of the figure that shows all slices can be found in the supplementary material.

Figure 5 and its animation found in the supplementary material give a side-by-side comparison of the directly measured components to those obtained from the XTT reconstruction. A slice of the reconstructed volumes is displayed for all scattering components and both methods. Here, both the XTT and registration-based datasets are normed to unity. Albeit conceptually very different, this comparison reveals that both methods yield remarkably similar results. This is a strong indication that both are equally valid and can be used for an orientation sensitive dark-field tomography.

**Figure 6:**
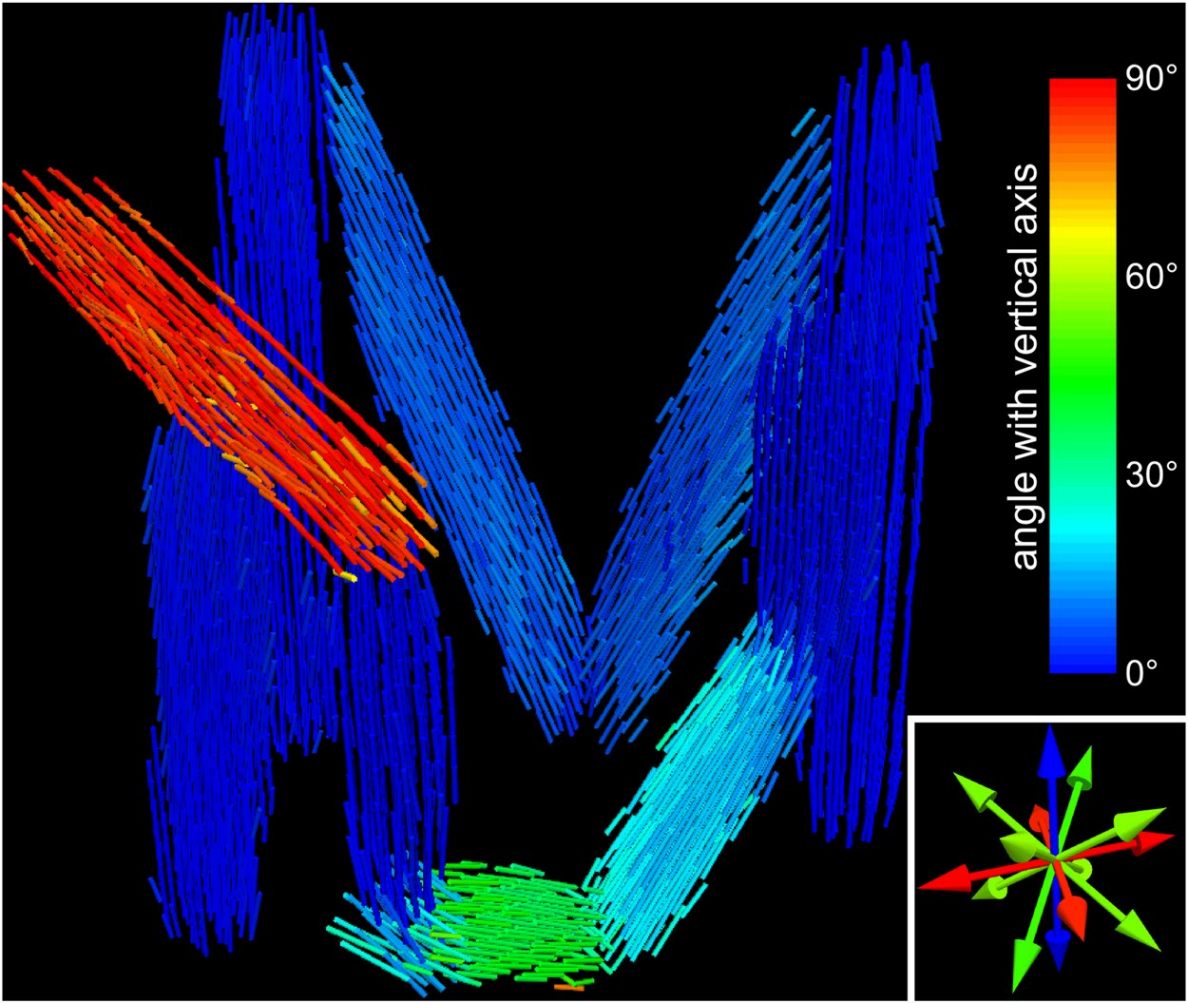
Visualization of the reconstructed structure orientation. From an ellipsoid fit to the scattering distribution in each voxel the smallest semi-principal axis is defined as structure orientation and visualized by little bars. Their respective colour indicates the angle between the local main structure orientation and the vertical axis. The main sensitivity orientations of the individual dark-field CT scans are shown in the inset.

Owing to this similarity, any subsequent data interpretation can be performed identically for XTT and the presented registration-based method. We fitted an ellipsoid to the directly reconstructed scattering distribution in each voxel. Bearing in mind that the scattering signal originates from fibrous structures we defined the structure orientation in each voxel as that of the smallest semi-principle axis of the ellipsoids^29^. This leaves us with a vector-field, visualized in Fig. 6 and the accompanying supplementary animation. We represent the main structure orientation at each point of the sample with small bars whose colour corresponds to their respective angle with the vertical axis. Structural information is displayed in every third voxel so that a clear visualization of the individual bars is possible. The main sensitivity orientations of the seven individual tomography datasets is shown in the inset. Although the probed orientations were chosen manually, the goal of an even distribution on the unit sphere was achieved. From this visualization it is clear that the reconstructed fibre orientation coincides well with the long axis of the individual wooden structures used to form the sample. This result demonstrates that the expected structure orientation can be well reconstructed from just a few sensitivity axes and subsequent ellipsoid fitting.

To conclude, we have presented an alternative way to perform orientation sensitive dark-field tomography using only well established reconstruction and image processing techniques. For this we changed the gratings of the interferometer used to acquire dark-field images to a horizontal position, which substantially improves the rotational invariance of the recorded scattering data and thus allows for a standard FBP reconstruction to be used. The presented method greatly simplifies the data acquisition as well as the reconstruction step. We showed that it is possible to directly measure the scattering components reconstructed with XTT by using a horizontal grating alignment. With a necessary volume registration step, the voxel-wise three-dimensional scattering distribution could be characterized, analogous to XTT. This had so far only been achieved by complex, highly elaborate reconstruction algorithms. The information contained in this multi-dimensional dataset was shown to contain detailed information about the underlying fibre structure for a wooden phantom. The possibility to substitute the complex reconstruction algorithms of XTT with several FBP reconstructions and subsequent registration of the volumes holds great potential. We believe that due to the decreased demand in highly specialized hardware and software, this alternative approach can be easily implemented in an existing grating interferometer. This will aid in making orientation sensitive dark-field tomography more accessible for future applications. Looking beyond grating-based X-ray dark-field imaging, the presented method has the potential to be applied to similar problems that attempt to reconstruct an oriented scattering distribution, e.g. neutron dark-field imaging.

## Methods

The measurements were performed at a symmetric three-grating experimental setup at the Technical University of Munich (TUM). Two gold absorption gratings with a period of 10 µm and height of $\approx$200 µm and $\approx$160 µm were used as G0 and G2, respectively. A third grating with 10 period and 8.6 high gold structures was used as $\pi$ phase-shifting G1 for a mean photon energy of 45 keV. The inter-grating distances were 919 mm.

X-rays were generated with an X-ray WorX 160-SE microfocus X-ray tube. A Varian PaxScan 2520DX detector with CsI scintillator and 127 µm pixel size, rebinned to an effective pixel size of 254 µm, was used to record images.

The sample was measured between G1 and G2 with a geometric magnification *M* = 1.14 and X-ray tube acceleration voltage of 60 p kVp and 125 W anode power. For each individual tomography set, 205 dark-field projections were recorded with 7 equidistant grating steps and 0.5 s exposure time per step.

Stepping analysis of the binned images was performed with an expectation maximization based algorithm. All reconstructions were performed using standard filtered back-projection. We used the commercially available software *Avizo Fire 8.0.1 - FEI, Hillsboro, Oregon, USA* for the volume registration step. The three-dimensional vector visualization was created with Paraview (www.paraview.org).

## Electronic supplementary material

Supplementary video 1

Supplementary video 2
